# Micro-Capillary Coatings Based on Spiropyran Polymeric Brushes for Metal Ion Binding, Detection, and Release in Continuous Flow

**DOI:** 10.3390/s18041083

**Published:** 2018-04-04

**Authors:** Aishling Dunne, Colm Delaney, Aoife McKeon, Pavel Nesterenko, Brett Paull, Fernando Benito-Lopez, Dermot Diamond, Larisa Florea

**Affiliations:** 1 Insight Centre for Data Analytics, National Centre for Sensor Research, Dublin City University, Dublin 9, Ireland; aishling.dunne58@mail.dcu.ie (A.D.); colm.delaney@dcu.ie (C.D.); aoifemckeon@rcsi.ie (A.M.); dermot.diamond@dcu.ie (D.D.); 2 Australian Centre for Research on Separation Science, and ARC Centre of Excellence for Electromaterials Science, Hobart, Tasmania 7001, Australia; pavel.nesterenko@utas.edu.au (P.N.); brett.paull@utas.edu.au (B.P.); 3 Analytical Microsystems & Materials for Lab-on-a-Chip (AMMa-LOAC) Group, Microfluidics Cluster UPV/EHU, Analytical Chemistry Department, University of the Basque Country UPV/EHU, Vitoria-Gasteiz 01006, Spain

**Keywords:** spiropyrans, polymer brushes, sensors, ROMP, coatings, photochromism, metal ion uptake and release, self-indicating system, micro-capillary

## Abstract

Micro-capillaries, capable of light-regulated binding and qualitative detection of divalent metal ions in continuous flow, have been realised through functionalisation with spiropyran photochromic brush-type coatings. Upon irradiation with UV light, the coating switches from the passive non-binding spiropyran form to the active merocyanine form, which binds different divalent metal ions (Zn^2+^, Co^2+^, Cu^2+^, Ni^2+^, Cd^2+^), as they pass through the micro-capillary. Furthermore, the merocyanine visible absorbance spectrum changes upon metal ion binding, enabling the ion uptake to be detected optically. Irradiation with white light causes reversion of the merocyanine to the passive spiropyran form, with simultaneous release of the bound metal ion from the micro-capillary coating.

## 1. Introduction

The photochromic properties of spirobenzopyrans were first discovered by Fischer and Hirshberg in 1952 [1]. Since then, spiropyrans have been studied for a wide range of applications including optical recording [2,3], photochromic lenses [4], dye-sensitised solar cells [5], light harvesting artificial membranes [6,7], sensors [8,9,10], and actuators [11,12,13,14,15], among others [16,17,18,19,20]. Upon irradiation with UV light, spiropyrans isomerise to the more polar, open merocyanine form. Metal ions can complex with the open merocyanine form, thereby influencing this isomerisation process. Conversely, irradiation with visible light results in a high concentration of the closed form, thereby releasing the metal ion. It is therefore possible to trigger metal ion binding by irradiation with UV light and to reverse this process through white light irradiation of the coloured complex. This regenerates the inactive spiropyran form and results in the release of metal ions [17]. 

The use of light to trigger the chelator offers unique opportunities, as the binding/releasing process is reversible and can be controlled externally in a non-invasive manner. We are particularly interested in this type of ‘switchable’ behaviour, as it enables a ‘4D’ character to be developed in which materials can respond to local stimuli (changes in local molecular environment, heat, light etc.) and switch between dramatically different modes of behaviour over time (the 4th dimension). Many of these behaviours, which are bio-inspired, have been previously used to generate micro-vehicles that can follow sources of chemical attractants by mimicking the movement of chemotactic organisms [21], or adaptive surfaces that can dramatically alter their physical and chemical properties in response to external stimuli [22,23].

In this paper, we present the metal ion binding capabilities of a norbornene-functionalised spiropyran monomer (SP) in solution, in addition to its photochromic behaviour in the crystal state and when polymerised to form 3D polymeric brushes on substrates. Moreover, through the integration of the beneficial characteristics of both miniaturised platforms and spiropyran photochromic dyes, a simple and innovative micro-capillary capable of switchable metal ion uptake and release has been realised that can simultaneously communicate its state (i.e., passive (non-binding); active (binding) and free; and active and populated). The functionalised micro-capillary model we have developed can therefore act as a photonically controlled self-indicating system for controlled metal ion uptake and release, operating in a continuous flow regime.

## 2. Experimental

### 2.1. Materials

7-Octenyltrichlorosilane (Gelest, Morrisville, PA, USA), 5-norbornene-2-carboxylic acid, exo- (Sigma-Aldrich, St. Louis, MO, USA), 1-(2-Hydroxyethyl)-3,3-dimethylindolino-6′-nitrobenzopyrylospiran (SP1) (TCI Europe, Zwijndrecht, Belgium), *N*,*N*′-dicyclohexylcarbo-diimide (DCC) (Sigma-Aldrich), 4-(dimethylamino)pyridine (DMAP) (Sigma-Aldrich), and Grubbs Generation-II catalyst (Sigma-Aldrich) were used as received. For the SP and poly(SP) synthesis, dry tetrahydrofuran and dry dichloromethane solvents were purchased from Sigma-Aldrich and used as received. Fused-silica micro-capillaries (100 μm ID, 375 μm OD) were purchased from Polymicro Technologies (Phoenix, AZ, USA). Acetonitrile (ACN) solvent used for solution and capillary studies was Sigma-Aldrich HPLC grade and was used without further purification. 

### 2.2. Synthesis of Spiropyran Norbornene Monomer (SP)

The spiropyran monomer (SP) was prepared from the reaction of exo-5-norbornyl carboxylic acid with SP1 in the presence of DCC and DMAP as described elsewhere [9]. After synthesis, the resulting red wax was purified using silica gel column chromatography and a solvent mixture of hexane: ethyl acetate (10:1). Crystals of SP, used to study the solid-state photochromism, were grown by slow evaporation from hexane: ethyl acetate (10:1). 

### 2.3. Synthesis of Spiropyran Polymeric Brushes (PolySP)

Si-ROMP was performed using a previously described method [9]. Prior to functionalisation, the micro-capillaries (internal diameter of 100 μm and 15 cm length) were washed with acetone and water. Following this, the fused silica surface was activated by passing a solution of 0.2 M NaOH for 30 min at a flow rate of 0.25 μL min^−1^ through the micro-capillary using a syringe pump, followed by a 0.2 M HCl solution for 30 min at the same flowrate. The micro-capillary was rinsed profusely with deionised water and dried under N_2_ stream after both the acid and base treatments. Next, the micro-capillary was flushed with a 0.1 M solution of 7-octenyl trichlorosilane in dry toluene for 90 min at a flow rate of 0.25 μL min^−1^. The micro-capillary was then washed with acetone, dried under a N_2_ stream, and left at room temperature for 24 h. Later, the micro-capillary was filled, in an inert atmosphere, with a 0.02 M solution of Grubbs Catalyst Second Generation in degassed CH_2_Cl_2_, sealed by rubber septa at both ends, and put in a water bath for 1 h at 45 °C. Following this, the catalyst-functionalised micro-capillary was thoroughly washed with degassed CH_2_Cl_2_ under inert atmosphere. Next, the micro-capillary was filed with SP monomer solution (0.5 M in degassed CH_2_Cl_2_), sealed at both ends using rubber septa, and put in a water bath at 50 °C for 1 h. The polymerisation was quenched by rinsing the micro-capillary with ethyl vinyl ether. Finally, the polySP polymeric-brushes functionalised micro-capillary was thoroughly washed with acetone to remove any physisorbed materials and dried under a N_2_ stream.

### 2.4. Light Source

Photo-conversion of the monomer solutions from SP to MC was achieved in a UVP CL-1000 Ultraviolet chamber using a wavelength of 365 nm at a power level of 3.5 mW cm^−2^ for 10 min (solutions) and 1–2 min (capillaries and crystals), respectively, to ensure full switching to the MC form. The white light irradiation was provided via a LMI-6000 LED Fiber Optic Illuminator obtained from Dolan-Jenner Industries and was used to switch the MC solutions and the polyMC coatings back to the closed SP and polySP forms, respectively. The vials/capillaries were placed at 2 cm from the light source and were irradiated for 2–3 min (solutions, crystals) and 10 min (capillaries), to ensure equilibrium interconversion. The maximum light output of the lamp was 780 Lumens, and the intensity control of the light output was fixed at 50%. 

### 2.5. Methods

UV-Vis spectroscopy of the SP solutions in the presence of different metal ions was performed using a Cary 50 spectrophotometer (Varian, Palo Alto, CA, USA). Microscopy images of the micro-capillaries and microscopy images and videos of the SP crystals were captured on an Aigo digital microscope (The Dolomite Centre Ltd., Royston, UK) equipped with auxiliary objectives to allow a magnification of 60×, 180× and 540×, respectively. UV-Vis spectroscopy was used to study the photo-binding and sensing of divalent metal ions to the poly(SP/MC) coatings while in different illumination conditions. The absorbance spectra were recorded using two fiber-optic light guides connected to a Miniature Fiber Optic Spectrometer (USB4000, Ocean Optics, Dunedin, FL, USA) and aligned using a cross-shaped cell (Supplementary Materials, Figure S1). The light source used was a DH-2000-92 BAL UV-NIR deuterium tungsten halogen source (Ocean Optics). The six metal ion solutions (in ACN) were passed through the micro-capillary at a constant flow rate of 0.5 µL min^−1^ using a syringe pump (PHD 2000 Syringe, Harvard Apparatus, Cambridge, MA, USA) and a glass Hamilton micro-syringe of 100 μL. The data from the spectrometer were processed using Spectrasuite software provided by Ocean Optics Inc. For clarity, each recorded absorbance spectrum was smoothed using Origin software with a Savitzky-Golay algorithm. The set-up used for metal ion detection after being photo-released from the micro-capillary is described in detail in the Supplementary Materials (ESI, Section S3).

The SP crystals were grown by slow evaporation from hexane: ethyl acetate (10:1 *v*/*v*) over several days to produce clear crystals.

## 3. Results and Discussion

### 3.1. Photochromism of Spiropyrans

Photochromism of spiropyran derivatives is reliant on the interconversion between the closed spiropyran (orthogonal) form and the open merocyanine (planar) form. For the most part, photochromism of spiropyran-derivatives has been largely studied in solution, but few derivatives are known to show photochromism in the crystalline state [24,25,26]. The common belief is that SP derivatives do not show solid state photochromism in crystals, though this has been disputed by Harada et al. [27] who demonstrated solid state photochromism in crystals for a range of spiropyrans and spirooxazines at low temperatures (−195 °C to −70 °C). 

**Figure 1:**
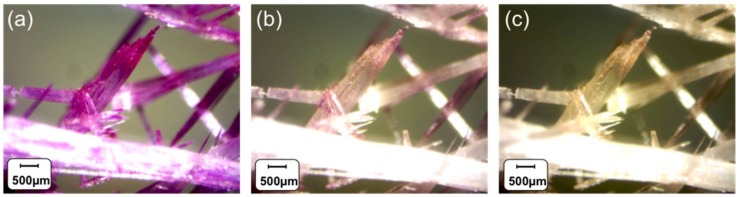
Crystals of the norbornene-spiropyran derivative (SP) exhibiting solid-state photoisomerisation under the following conditions: (**a**) 1 min UV irradiation, (**b**) additional 1 min white light irradiation, and (**c**) additional 2 min white light irradiation. The strong purple colour in (**a**) is indicative of significant formation of the merocyanine isomer, whereas the lighter purple colour in (**b**) indicates co-existence of both isomers. The colourless crystals (**c**) indicate that the system has largely reverted to the spiropyran isomer.

However, the SP monomer synthesised in this research exhibits rapid and reversible room temperature photochromism in the crystal state upon UV/white light irradiation (Video S1). Upon UV light (365 nm) exposure, the colour of the crystals rapidly changes towards purple (Figure 1a; 1 min UV irradiation). Progressive reversal of this process is indicated by decolouration produced by illumination with white light (Figure 1b, 1 min, and Figure 1c, additional 2 min). This effect is observed at room temperature (20 °C). Although the determination of the crystal structure in the two forms was attempted, as in previously reported cases [27], this was unfruitful, most likely because of the disorder arising from the co-existence of both SP and MC forms in the crystal lattice. Further investigation will be carried out to ascertain if photo-conversion takes place at crystal sites under lattice control or is restricted to crystal defects. 

### 3.2. Metal Ion Binding—Solution Studies

Solution studies have shown that the open MC form of the spiropyran norbornene functionalised monomer can bind certain metal ions in solution. An integral characteristic of spiropyran is the photo-cleavage of the C_spiro_-O bond, which occurs during UV illumination, to generate the merocyanine form. The merocyanine possesses a negatively charged phenolate oxygen which serves as a potential site for cation binding [28,29,30,31,32]. In this context, there have been several research studies on SP-derivatives for metal ion binding, predominantly for divalent metal ions such as Zn^2+^ [32,33], Co^2+^ [10,33,34], Cu^2+^ [33,34], Ni^2+^ [30,33], and Cd^2+^ [35], in which binding generally happens in a 2:1 ratio of merocyanine to M^2+^, due to the coordination of the divalent metal ion by two MC phenolate anions [35]. Several research groups have attempted to increase the number of binding sites and achieve a 1:1 binding ratio (merocyanine: M^2+^) through variation of the R group in the eighth position of the benzopyran ring to include methoxy [34] or allyl substituents [34], or by functionalising the indolic nitrogen with carboxylic acid [36], ester [37], or hydroxyl-terminated moieties [34]. These groups can promote complex formation by offering additional sites to the merocyanine phenolate anion for stabilisation of the metal centre, resulting in a thermodynamically stable MC:M^2+^ complex [32]. However, the metal-ligand interaction is usually weak enough to allow disruption of the complex by the photo-induced ring closing reaction. Therefore, SP derivatives can be used as reversible chelators for metal ions.

**Figure 2:**
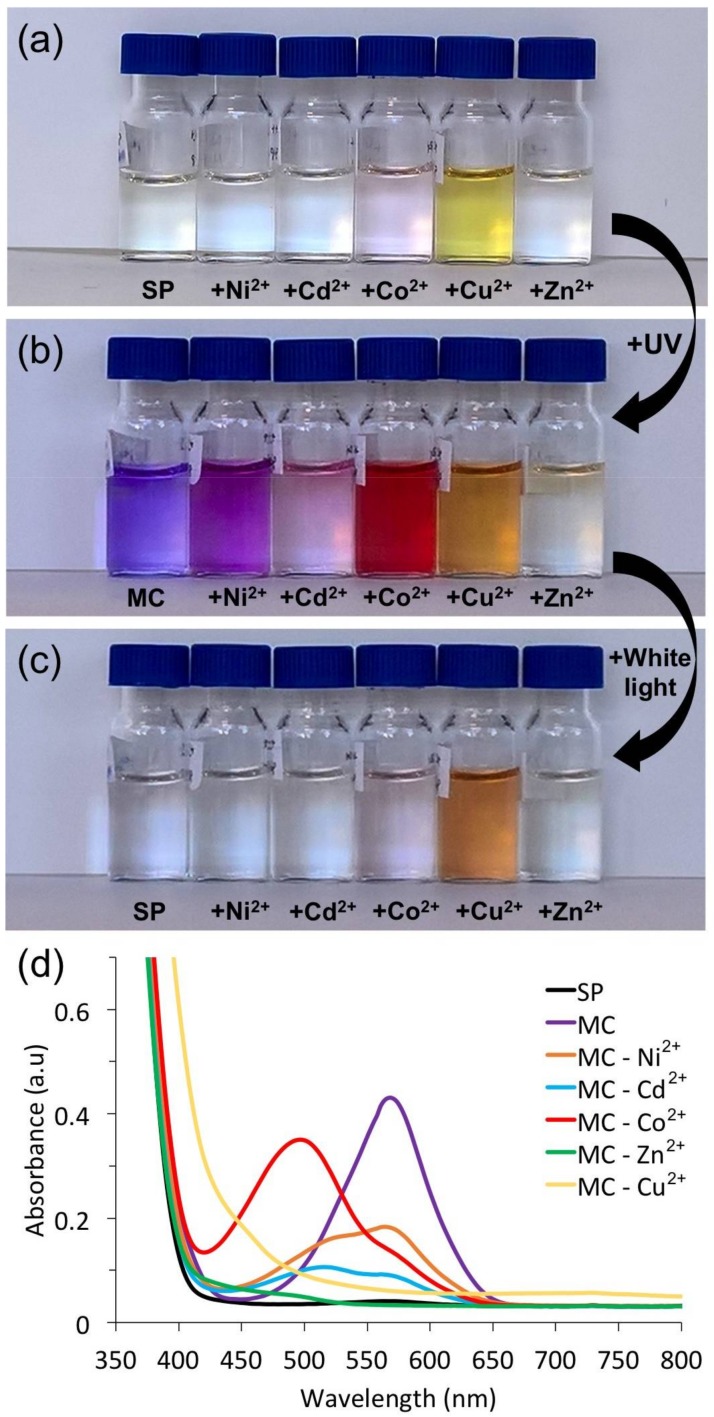
Images (**a**–**c**) and UV-Vis spectra (**d**) of the SP solutions in ACN (1.5 × 10^−3^ M) in the presence of various divalent metal ions (molar ratio SP:M^2+^ 2:1); (**a**) initial solutions before illumination; (**b**) after 10 min of UV light illumination; (**c**) after 10 min of white light illumination; (**d**) UV-Vis spectra of solutions shown in (**b**).

The SP norbornene functionalised spiropyran ester, used in this study, has two potential binding sites for the metal ion; the phenolic oxygen of the MC form, and carbonyl oxygen of the ester side chain, and could potentially allow for both 1:1 and 2:1 (MC:M^2+^) binding ratios. The MC−M^2+^ complexes have been successfully characterised in solution using UV-Vis spectroscopy. In the presence of the MC form, certain metal ions exhibit a unique colorimetric response, due to formation of the metal ion complexes (Figure 2a–c). The colourless, ring-closed spiropyran (SP) shows no absorption in the visible region. After irradiation of the solution with UV light (2 min), the ring-opened merocyanine (MC) form is clearly visible, characterised by an intense absorption at ~570 nm, specific to the zwitterionic MC form. Upon complexation with Ni^2+^, Cd^2+^, Co^2+^, Zn^2+^, and Cu^2+^ ions, the absorption band is shifted to lower wavelengths. The MC:Ni^2+^ complex yields a small hypsochromic shift of 40 nm (λ_max_ ≈ 530 nm. The MC:Co^2+^ and MC:Zn^2+^ complexes exhibit blue shifts of 70 nm (λ_max_ ≈ 500 nm) and 90 nm (λ_max_ ≈ 460 nm), respectively. Shifts in the λ_max_ can be attributed to the disruption of planarity in trans-MC [18]. In the case of some of the metal ions (Ni^2+^, Co^2+^, Cd^2+^), as seen in Figure 2d, the characteristic MC absorbance band still remains. This suggests that under the same experimental conditions (1.5 × 10^−3^ M SP in ACN, 2:1 molar ratio SP:M^2+^, and identical illumination conditions), the MC presents different binding affinities for different metal ions. The co-existence of both absorbance features characteristic of the free MC and the MC:M^2+^ complex is evidence of an equilibrium between the two forms. Irradiation of the solutions with white light caused the disappearance of the band characteristic to MC and MC:M^2+^, indicating reversible binding for all cases except Cu^2+^ (Figure 2c). While this result is somewhat surprising, it has been previously demonstrated by Natali et al. [33] that the merocyanine:Cu^2+^ interaction is rather “curious”, as it can lead to the formation of stable SP dimers mediated by Cu^2+^. We believe a similar symmetric dimer is obtained in the case of this SP in the presence of Cu^2+^ analogous to that suggested by Natali et al. [33].

**Figure 3:**
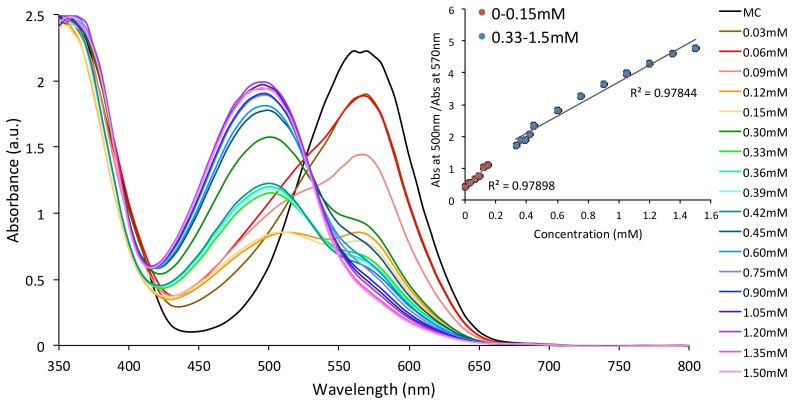
Absorbance changes of the SP solutions in ACN (1.5 × 10^−3^ M) in the presence of increasing concentrations of Co^2+^ (0–1.5 mM) after irradiation with UV light; Inset shows the linear dependence of the ratio of the absorbance at λ_max_ ≈ 500 nm (MC:Co^2+^) and the absorbance at λ_max_ ≈ 570 nm (MC) as a function of concentration of Co^2+^ for concentration ranges 0–0.15 mM and 0.33–1.5 mM.

To evaluate the potential of the MC form of the SP norbornene functionalised spiropyran ester as a quantitative probe for divalent metal ions, MC was subjected to UV-Vis titrations with increasing concentrations of Co^2+^ and Cu^2+^, respectively. Figure 3 shows the absorbance spectra of MC (1.5 × 10^−3^ M SP in ACN) in the presence of increasing concentrations of Co^2+^. As observed, the absorbance band specific to the MC form is seen to decrease with increasing Co^2+^ (0–0.15 mM), with the simultaneous appearance of a new shoulder at ~500 nm. Increasing the concentration of Co^2+^ even further (0.15 mM–1.5 mM) causes the appearance of the MC:Co^2+^ band centred at λ_max_ ≈ 500 nm, which induced a color change from purple (MC) to red (MC:Co^2+^). A linear dependence of the ratio of the absorbance at λ_max_ ≈ 500 nm (MC:Co^2+^) and the absorbance at λ_max_ ≈ 570 nm (MC) as a function of concentration of Co^2+^ for concentration ranges 0–0.15 mM and 0.33–1.5 mM (Figure 3, inset) was observed. 

**Figure 4:**
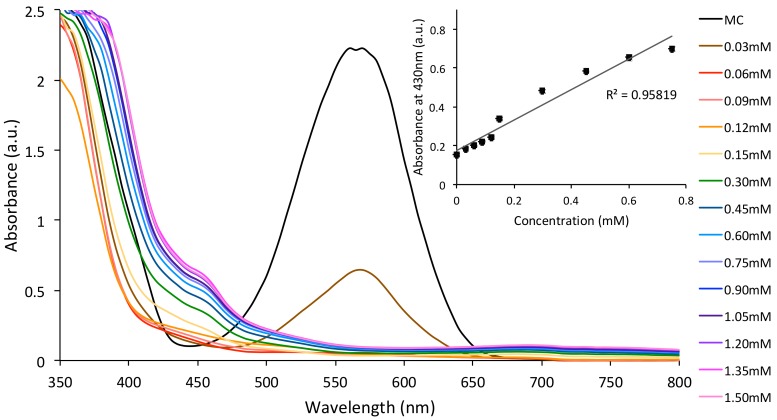
Absorbance changes of the SP solutions in ACN (1.5 × 10^−3^ M) in the presence of increasing concentrations of Cu^2+^ (0–1.5 mM) after irradiation with UV light; Inset shows the linear dependence of the absorbance at λ_max_ ≈ 430 nm (MC:Cu^2+^) as a function of Cu^2+^ concentration (0–0.8 mM).

Similarly, the spectrophotometric titration of MC (1.5 × 10^−3^ M SP in ACN) with Cu^2+^ (0–1.5 mM) was also investigated under the same conditions (2 min of UV irradiation). In this case, the absorbance band specific to MC (λ_max_ ≈ 570 nm) disappeared completely once the concentration of Cu^2+^ was increased upon 0.03 mM and a new absorbance band specific to MC:Cu^2+^ was observed, centred at 430 nm. A linear dependence of this absorbance band (λ_max_ ≈ 430 nm) as a function of Cu^2+^ concentration was observed (Figure 4, inset). No noticeable difference in the MC binding behaviour was observed when the Co^2+^ or Cu^2^ solutions containing 10^−3^ M Na^+^. This result is expected, as MC dyes are not known to bind monovalent metal ions.

### 3.3. Metal Ion Binding—Micro-Capillary Studies

**Figure 5:**
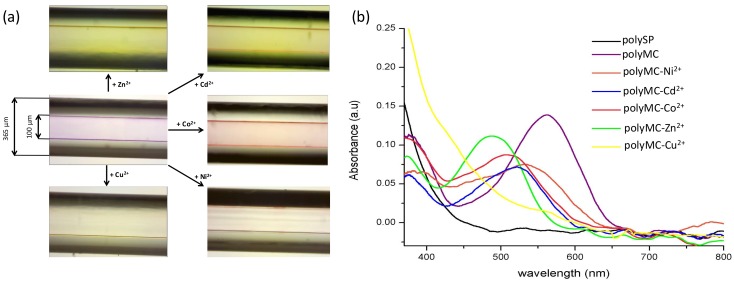
Images (**a**) and absorption spectra (**b**) of the coated micro-capillaries when solutions of different metal ions in ACN (10^−3^ M) are passed through the micro-capillary after irradiation for 1 min with UV light.

**Figure 6:**
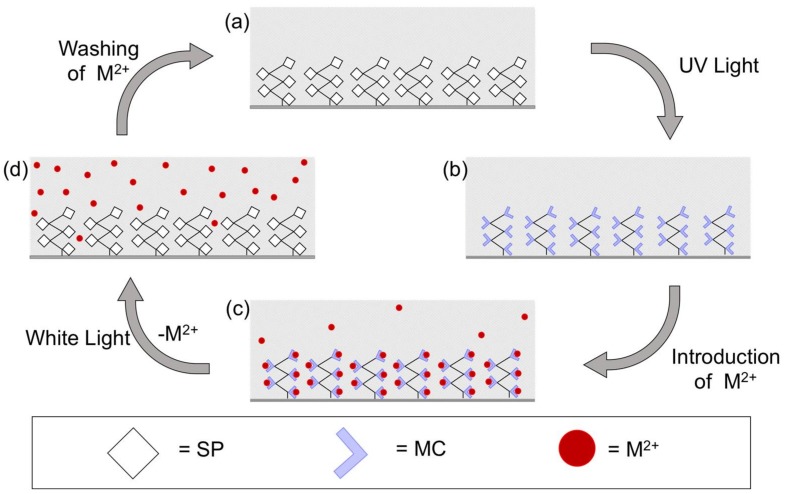
Schematic representation of the metal ion binding, sensing, and release cycle in the SP polymeric brushes coated micro-capillary. (**a**) polySP coated micro-capillary before irradiation; (**b**) formation of polyMC upon UV irradiation; (**c**) binding of M^2+^ to polyMC coating; (**d**) photo-controlled release of M^2+^ upon white light irradiation and regeneration of polySP coating.

Coating of the glass micro-capillaries was performed using ring-opening metathesis polymerisation, as described in the experimental section, to produce a 2–3 μm homogenous coating (Figure S2) comprised of colourless spiropyran (SP) homopolymeric brushes. We have previously characterised such polymeric brushes [9], in addition to their UV-induced photochromism [9] and solvatochromic properties [10]. Figure 5 shows the colour of the micro-capillary post UV light irradiation in the presence of divalent metal ions and the corresponding UV-vis spectra. Upon irradiation with UV light, the SP form (non-binding) (Figure 6a) is converted to the polar MC form that exhibits metal ion chelation capabilities (Figure 6b). When certain divalent metal ion solutions (e.g., Cu^2+^, Co^2+^, Cd^2+^, Ni^2+^, Zn^2+^) are passed through the coated micro-capillary in a continuous flow, metal ions are bound by free MC sites (Figure 6c) in the capillary coating through the spontaneous formation of polyMC:M^2+^, signalled by clearly visible changes in the colour of the micro-capillary (Figure 5). Subsequent irradiation of the micro-capillary with white light causes the MC:M^2+^ to revert to the SP form and the bound metal ion is released (Figure 6d). Simultaneously, the micro-capillary reverts to colourless and the passive SP-coating is regenerated (Figure 6a). The change in colour of the micro-capillary upon UV irradiation in the absence or presence of the different metal ion solutions under continuous flow (0.5 µL min^−1^) was recorded using the set-up described in Figure S3. The corresponding spectra are presented in Figure 5b. UV-Vis spectroscopy studies of the SP polymer (polySP) brush films on the micro-capillary revealed that UV irradiation leads to a strong absorbance in the visible region centred at approximately 565 nm, corresponding to conversion of the SP-functionalised norbornene polymer to the MC form (polyMC). As in the case of the monomer in solution, this absorbance λ_max_ is shifted to lower wavelengths in the presence of the metal ions in the order: MC (λ_max_ ≈ 565 nm) > MC:Ni^2+^(λ_max_ ≈ 534 nm) > MC:Cd^2+^(λ_max_ ≈ 522 nm) > MC:Co^2+^(λ_max_ ≈ 508 nm) > MC:Zn^2+^(λ_max_ ≈ 489 nm) > MC:Cu^2+^(λ_max_ ≈ 430 nm). The fact that the absorption characteristic of the MC unbound form does not appear in any of the polyMC:M^2+^ spectra, indicates that polyMC is extensively involved in the complex formation. A broadening of the absorption bands is also observed compared to the solution studies (Figure 2d). The coated micro-capillary represents a self-indicating, photo-controlled system that can report its status—passive, active (free), bound with metal ion through changes in colour/absorbance; and to some extent, it is possible to distinguish which metal ion is bound from differences in the visible absorbance spectrum. As in the case of solution studies, it was observed that the binding process was reversible for each of the metal ions (Figure S4) except for Cu^2+^. 

**Figure 7:**
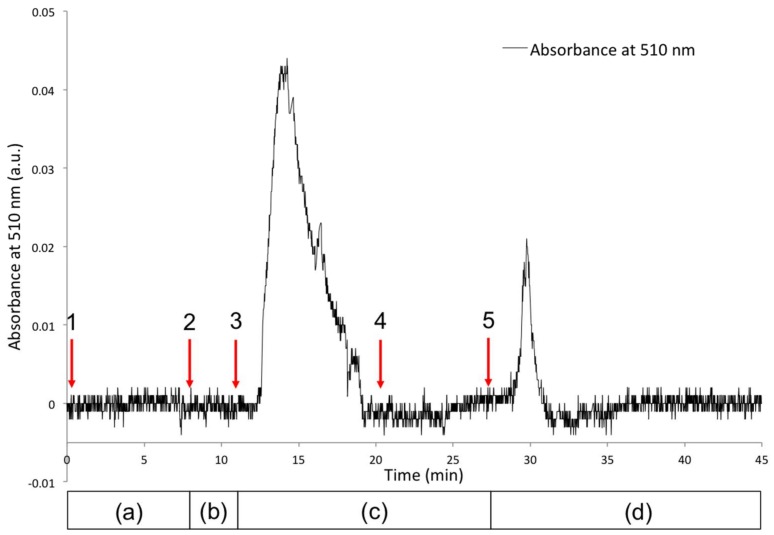
Absorbance at 510 nm recorded on a USB400 spectrometer over time, using the set-up depicted in Figure S7. Five stages (1–5) are identified as described in the text; The increase of the absorbance band centred at 510 nm indicates the presence of PAR-Co^2+^ complex; Bottom bar indicates the state of the functionalised micro-capillary as per Figure 6: (a) polySP coated micro-capillary before irradiation; (b) formation of polyMC upon UV irradiation; (c) binding of Co^2+^ to polyMC coating; (d) photo-controlled release of Co^2+^ upon white light irradiation and regeneration of polySP coating.

Co^2+^ was selected for further study on the basis of its very clear spectral shift from free MC to MC:M^2+^. In addition to the spectral changes inherent to the ion binding and release observable from the coating in the capillary, detection of free Co^2+^ released from the poly(SP) functionalised micro-capillary upon white light irradiation was evaluated using a post column reaction with 4-(2-pyridylazo)resorcinol (PAR). PAR is a well-known chelating reagent, offering multiple coordination possibilities for metal ions [38,39] through a pyridyl nitrogen, azo group and o-hydroxyl group, as shown in Figure S5. This results in an easily identifiable PAR:Co^2+^ coloured complex with a λ_max_ at ~510 nm, as shown in the UV-Vis spectra in Figure S6. The set-up used for recording the absorbance at 510 nm before and after irradiation of the poly(MC) functionalised micro-capillary with white light (release) is shown in Figure S7. Figure 7 shows the monitoring of the absorbance at 510 nm (characteristic for the PAR:Co^2+^ coloured complex) as a function of time. Several stages (1–5) are identified (Figure 7), in relation to the state of the functionalised micro-capillary (Figure 6), as described below:

1. Passive form (polySP) in the presence of solvent; capillary is colourless, no Co^2+^ present; low baseline signal at 510 nm (Figure 6a).
2. UV source ON; passive polySP converted to active binding polyMC form; capillary has a purple colour (Figure 5a); no Co^2+^ present; low baseline signal at 510 nm (Figure 6b).
3. Co^2+^ is injected in the mobile phase; capillary indicates binding by colour change to a reddish colour (Figure 5a and Figure 6c); large peak at 510 nm is due to the detection of unbound Co^2+^ passing through the micro-capillary.
4. Switch back to solvent without Co^2+^; signal returns to baseline indicating unbound Co^2+^ has been removed. Capillary still has a reddish colour suggesting bound Co^2+^ is present in the coating (Figure 6c).
5. White light source is turned ON; coating reverts to colourless (polySP) passive form; bound Co^2+^ is simultaneously released (Figure 6d) and detected as the peak at ca. 30 min.

This experiment confirms the release of Co^2+^ from the micro-capillary upon white light irradiation. In the case of Cu^2+^, the micro-capillary maintained its yellow colour after the binding event, despite white light irradiation. This suggests that the dimer formation mentioned previously is also occurring in the polymeric brushes. 

## 4. Conclusions

Herein, we have demonstrated the photochromism of a norbornene-functionalised spiropyran derivative and its ability to reversibly bind divalent metal ions present in solution, accompanied by significant variations in the visible absorbance spectrum. Micro-capillaries were coated with spiropyran polymer brushes using ROMP chemistry, and the resulting flow system was found to exhibit self-indication of status (passive, active, metal-ion bound) through a colorimetric response. In addition, this metal ion uptake and release behaviour is photo-controlled using external light sources. Although photo-controlled metal ion detection using spiropyran derivatives has been previously demonstrated by us [17] and others [33,34,35,36,37], this represents the first example in which photo-controlled uptake and release has been demonstrated within a micro-capillary system using polymeric brushes, in a continuous flow regime. Although, without doubt, there are many specific sensor molecules for quantitative metal ion detection, the ability to achieve a self-indicating flow-system, capable of photo-controlled binding and release, may prove to be of particular importance for the integration of advanced bioinspired functionalities in microfluidic systems. The use of light to trigger the chelator offers unique opportunities that minimise waste generation and power requirements. In addition, advances in the integration of LED (light emitting diode) sources in the system hold promise for the production of low cost miniaturised systems. 

## Supplementary Materials

The following are available online at http://www.mdpi.com/1424-8220/18/4/1083/s1, Figure S1: Schematic representation of the polySP polymeric brush structure and functionalised micro-capillary; Figure S2: Scanning Electron Microscopy image of the polySP polymeric brushes functionalised micro-capillary; Figure S3: Set-up used to study the absorbance spectra of the micro-capillary when M^2+^ solutions (in ACN) are passed through the micro-capillary in continuous flow. The set-up is composed of a two fiber-optic light guides connected to a light source and a Miniature Fiber Optic Spectrometer (USB4000, Ocean Optics) and aligned using a cross-shaped cell. The M^2+^ solution (in ACN) is passed through the micro-capillary using a syringe pump; Figure S4: Microscopy photos of a section of a micro-capillary modified with spiropyran polymer brushes (polySP) before (left) and after irradiation for 20 s with UV light (middle) followed by the addition of Co^2+^ (right). The micro-capillary returns to colourless (due to the conversion of the polyMC to polySP) after irradiation with white light for 1 min, resulting in the release of Co^2+^ ions; Figure S5: Chemical structures of 4-(2-pyridylazo) resorcinol (left) and metal complexed 4-(2-pyridylazo) resorcinol (right); Figure S6: Absorbance spectra of the chelating reagent (PAR) and its Co^2+^ complex; Figure S7: Scheme of the set-up used for the determination of metal ions photo-released from the polySP modified micro-capillary using PAR. Step 1 (irradiation of the spiropyran modified micro-capillary with UV light) and 5 (irradiation of spiropyran modified micro-capillary with white light) are depicted in the scheme; Figure S8: Absorbance at 510 nm recorded on a USB400 spectrometer using the set-up depicted in Figure S7 during experimental steps 1–6. The increase of the absorbance band centred at 510 nm indicates the presence of PAR-Co^2+^ complex; Video S1: Real time colour change of the spiropyran norbornene monomer crystals under different illumination conditions.
