# Supplementary material for: Micro-Capillary Coatings Based on Spiropyran Polymeric Brushes for Metal Ion Binding, Detection, and Release in Continuous Flow
Source: Sensors (Basel). 2018 Apr 4;18(4):1083. doi: 10.3390/s18041083 (PMC5949026; doi:10.3390/s18041083)
Supplement: Supplementary file 1 [file sensors-18-01083-s001.pdf]

Supplementary Information

Micro-capillary Coatings Based on Spiropyran Polymeric Brushes for Metal Ion Binding, Detection and Release in Continuous Flow

Aishling Dunne [1], Colm Delaney [1], Aoife McKeon [1,†], Pavel Nesterenko [2], Brett Paull [2], Fernando Benito-Lopez [3,*], Dermot Diamond [1] and Larisa Florea [1,*]

[1] Insight Centre for Data Analytics, National Centre for Sensor Research, Dublin City University, Dublin 9, Ireland; aishling.dunne58@mail.dcu.ie (A.D.); colm.delaney@dcu.ie (C.D.); aoifemckeon@rcsi.ie (A.M.); dermot.diamond@dcu.ie (D.D.)

[2] Australian Centre for Research on Separation Science, and ARC Centre of Excellence for Electromaterials Science, Hobart, Tasmania 7001, Australia; pavel.nesterenko@utas.edu.au (P.N.); brett.paull@utas.edu.au (B.P.)

[3] Analytical Microsystems & Materials for Lab-on-a-Chip (AMMa-LOAC) Group, Microfluidics Cluster UPV/EHU, Analytical Chemistry Department, University of the Basque Country UPV/EHU, Vitoria-Gasteiz 01006, Spain

Correspondence: fernando.benito@ehu.eus (F.B.-L.); larisa.florea@dcu.ie (L.F.); Tel.: +353-01-700-6009 (L.F.); +34-945-01-3045 (F.B.-L.)

† Current Address: Centre for Synthesis and Chemical Biology, Department of Pharmaceutical and Medicinal Chemistry, Royal College of Surgeons in Ireland, 123 St. Stephens Green, Dublin 2 D02 YN77, Ireland.

Table of Contents:

S1: polySP polymeric brushes functionalised micro-capillary
S2: Set-up for absorbance measurements of micro-capillaries
S3: Photo-induced binding and releasing of metal ions
S4: Videos
S5: References

S1. polySP polymeric brushes functionalised micro-capillary

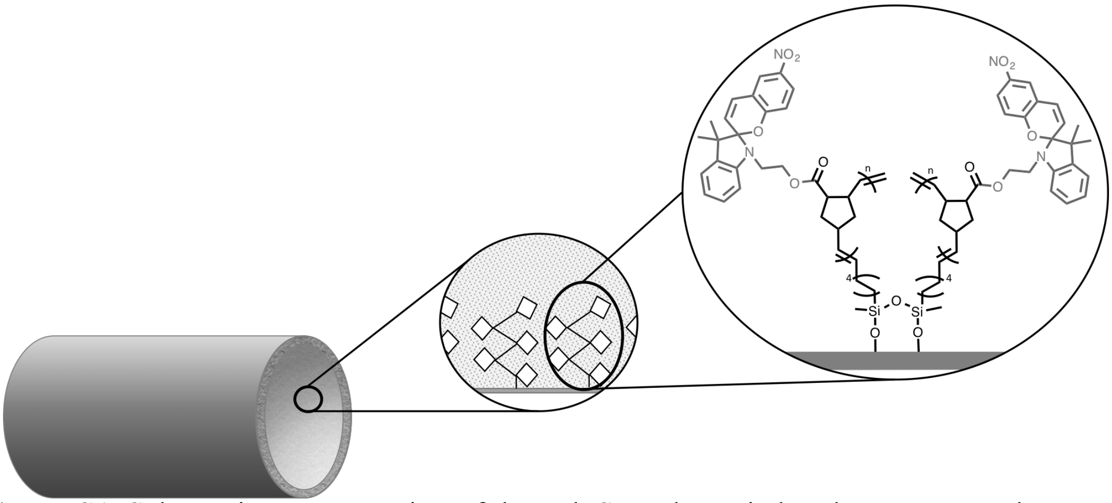

Figure S1. Schematic representation of the polySP polymeric brush structure and functionalised micro-capillary.

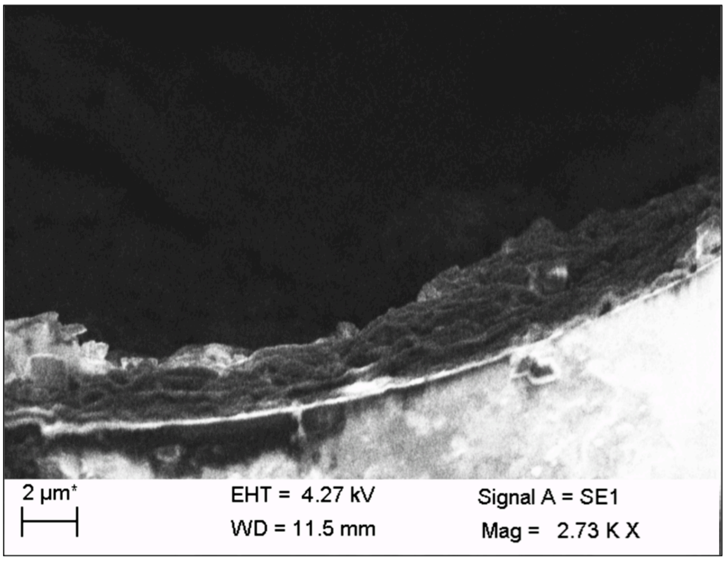

Figure S2. Scanning Electron Microscopy image of the polySP polymeric brushes functionalised micro-capillary.

S2. Set-up for absorbance measurements of micro-capillaries

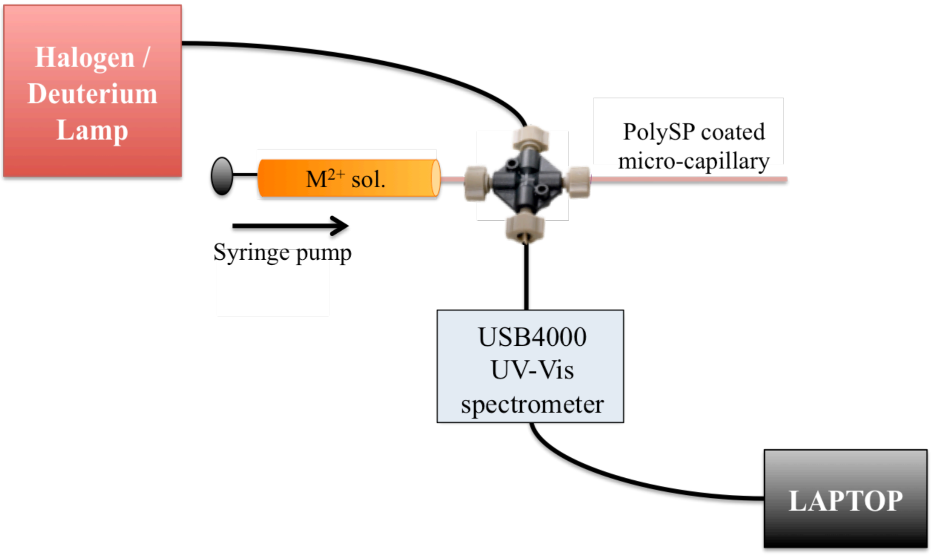

Figure S3. Set-up used to study the absorbance spectra of the micro-capillary when M^{2+} solutions (in ACN) are passed through the micro-capillary in continuous flow. The set-up is composed of a two fiber-optic light guides connected to a light source and a Miniature Fiber Optic Spectrometer (USB4000, Ocean Optics) and aligned using a cross-shaped cell. The M^{2+} solution (in ACN) is passed through the micro-capillary using a syringe pump.

S3. Photo-induced binding and releasing of metal ions

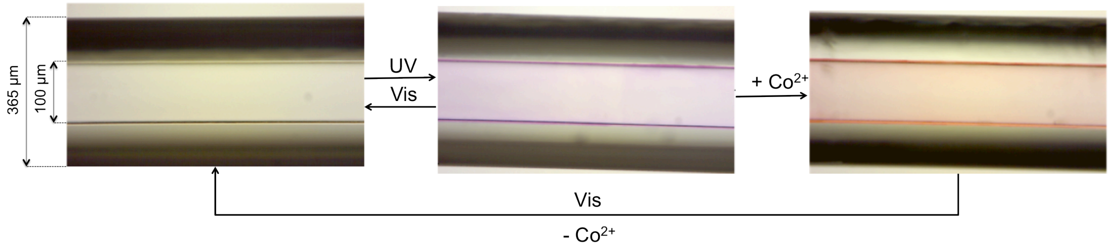

Figure S4. Microscopy photos of a section of a micro-capillary modified with spiropyran polymer brushes (polySP) before (left) and after irradiation for 20 s with UV light (middle) followed by the addition of Co^{2+} (right). The micro-capillary returns to colourless (due to the conversion of the polyMC to polySP) after irradiation with white light for 1 min, resulting in the release of Co^{2+} ions.

In order to prove the release of the bound metal ion from the SP-polymer brushes coated micro-capillary through irradiation with white light, the release of metal ion was demonstrated in the case of Co^{2+} through detection post modified micro-capillary using a chelating reagent, 4-(2-pyridylazo)resorcinol (PAR). PAR can coordinate to

metal ions through a heterocyclic nitrogen group, azo group, and *o*-hydroxyl group, as shown in Figure S5[1-3].

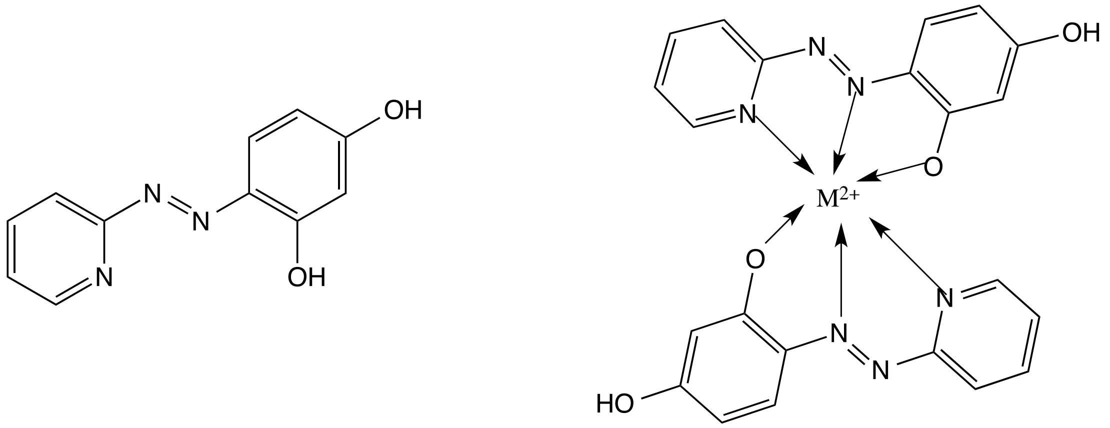

Figure S5. Chemical structures of 4-(2-pyridylazo) resorcinol (left) and metal complexed 4-(2-pyridylazo) resorcinol (right).

Firstly, the absorbance spectra of the chelating reagent (PAR) and its Co^{2+} complex were recorded (Figure S6) by passing a solution of PAR (1 mM) and PAR-Co^{2+} (PAR: Co^{2+} 1:1) through an unmodified glass micro-capillary at 2 μL min^{-1}. The spectra (Figure S6) show the typical absorbance bands corresponding to PAR (black) and PAR-Co^{2+} (red). The absorbance maximum for PAR-Co^{2+} was recorded at ~ 510 nm.

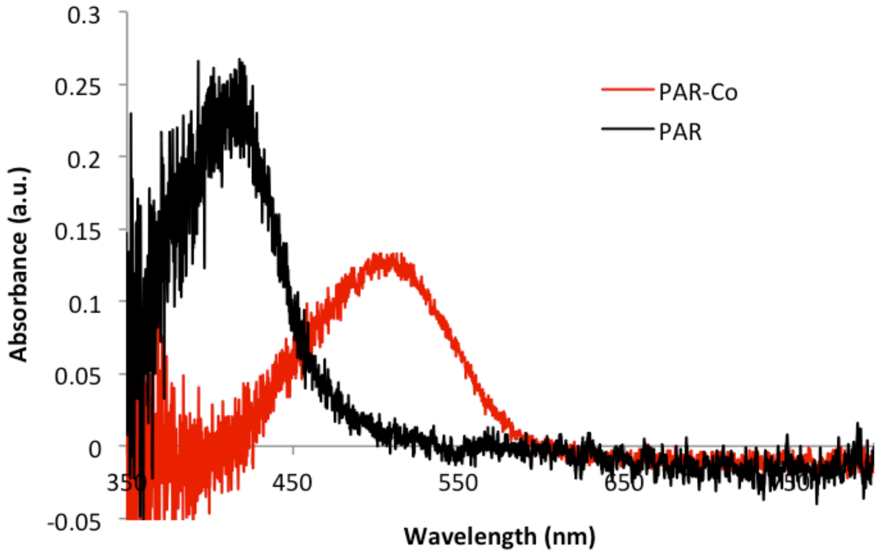

Figure S6. Absorbance spectra of the chelating reagent (PAR) and its Co^{2+} complex.

For the detection of the photo-released Co^{2+}, the previous set-up (Figure S3) was modified (Figure S7) to include the injection of Co^{2+} solution in ACN (1 mM), and the following steps were undertaken:

1. The pump (left) was turned on (flow rate = 20 µL min^{-1}; mobile phase = ACN).
2. The syringe pump (right) was turned on (flow rate = 20 µL min^{-1}; mobile phase = post column reagent PAR 0.1 M).
3. The polySP modified micro-capillary was irradiated with UV light for 20 s.
4. Co^{2+} solution (1 mM) from the injection loop was injected in the system at a flow rate of 20 µL min^{-1} for approximately 5 min.
5. When all the expected Co^{2+} solution left the detection area, both pumps (ACN and PAR) were turned *OFF* and the while light was turned *ON*.
6. After about 5 min, both pumps (ACN and PAR) were turned back *ON*.
7. The absorbance at λ_{max} specific for PAR-Co^{2+} (510 nm) was recorded during the whole experiment (steps 1-6) and plotted in Figure S8.

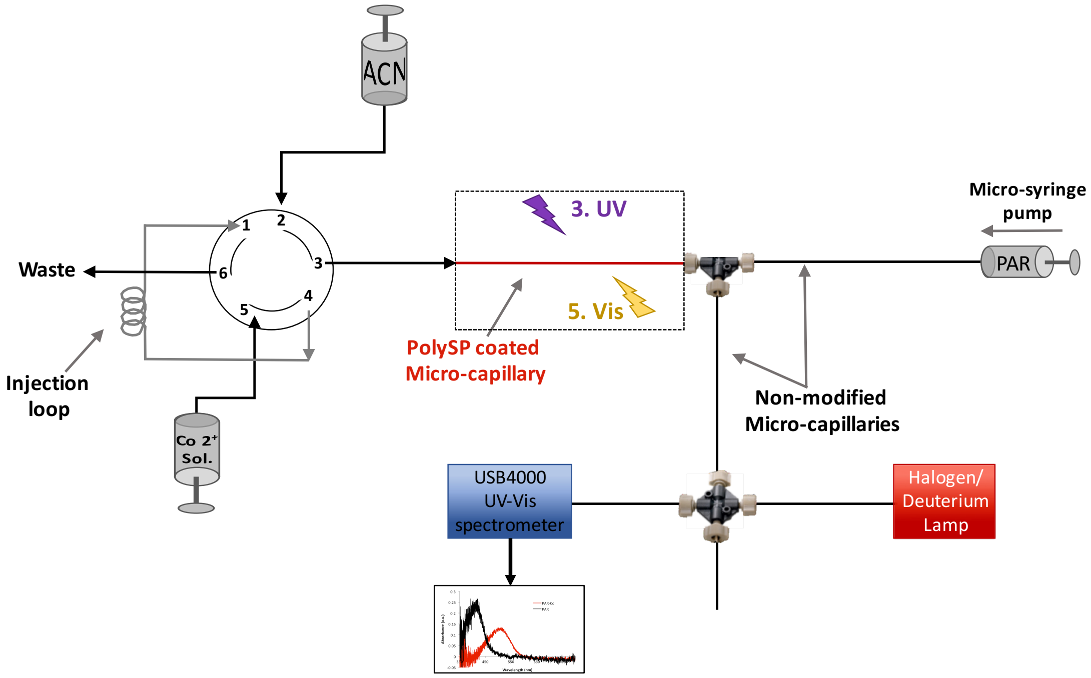

Figure S7. Scheme of the set-up used for the determination of metal ions photo-released from the polySP modified micro-capillary using PAR. Step 3 (irradiation of the spiropyran modified micro-capillary with UV light) and 5 (irradiation of spiropyran modified micro-capillary with white light) are depicted in the scheme.

It is expected that, after the irradiation of the micro-capillary with white light (step 5), the Co^{2+} ions will be released and then, with both pumps turned ON, the two confluent flows will react and PAR-Co^{2+} will be formed. When reaching the detection area, PAR-Co^{2+} will generate a change in the absorption spectra, generating a new absorbance band at 510 nm. This absorbance band (Figure S8) was recorded during the experiment (steps 1 to 6) and shows an increase in the absorbance band at 510 nm when both the PAR flow (step 2) and Co^{2+} flow (step 4) are turned ON. When the Co^{2+} flow is turned OFF (step 5), a decrease in the band at 510 nm is observed until this reaches an absorbance of ~0 a.u. indicating that all Co^{2+} has exited the detection area. Following this, the PAR flow is also switched OFF and the SP-M polymeric brushes functionalised micro-capillary is irradiated with white light for 5 minutes. Finally, the ACN and PAR flows are switched ON. This causes an increase in the band at 510 nm (Figure S8, step 6) indicating that indeed Co^{2+} was released upon white light irradiation from the modified micro-capillary.

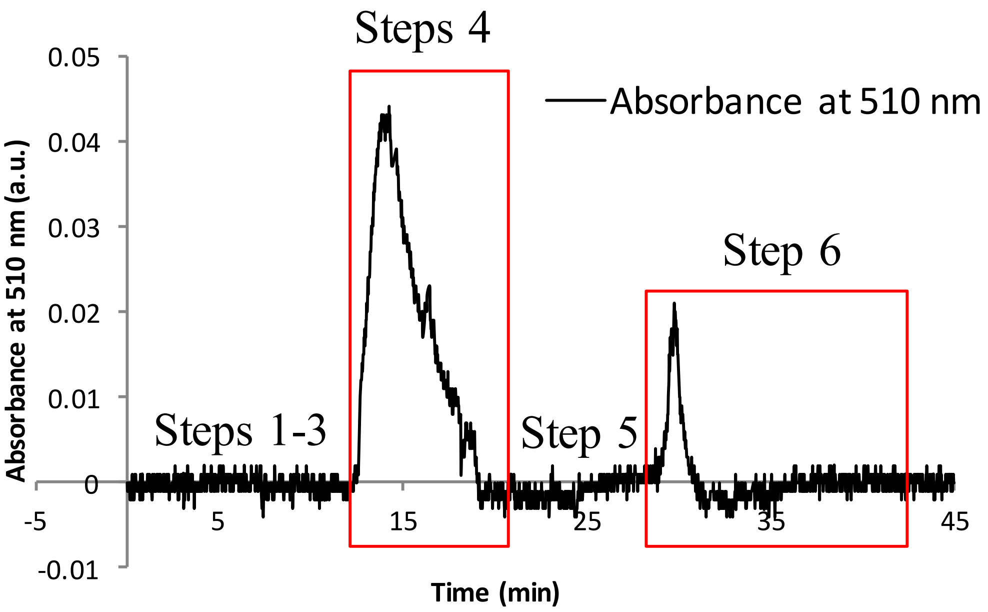

Figure S8. Absorbance at 510nm recorded on a USB400 spectrometer using the set-up depicted in Figure S7 during experimental steps 1-6. The increase of the absorbance band centred at 510nm indicates the presence of PAR-Co^{2+} complex.

S4. Videos

Video S1 shows in real time the colour change of the spiropyran norbornene monomer crystals under different illumination conditions. In the video, the UV light was turned ON at 0:45 and switched OFF after ~ 2 min (time 2:49), followed by ~ 3 min of white light irradiation (white light ON at 5:13 and switched OFF at 8:21). The video was recorded on a benchtop Aigo digital Microscope GE5, at a magnification of 180x.

S5. References

1. Ghasemi, J.; Niazi, A.; Maeder, M. Spectrophotometric studies on the protonation and nickel complexation equilibria of 4-(2-pyridylazo) resorcinol using global analysis in aqueous solution. *Journal of the Brazilian Chemical Society* **2007**, *18*, 267-272.
2. Ghasemi, J.; Peyman, H.; Meloun, M. Study of complex formation between 4-(2-pyridylazo) resorcinol and Al^{3+}, Fe^{3+}, Zn^{2+}, and Cd^{2+} ions in an aqueous solution at 0.1 m ionic strength. *J. Chem. Eng. Data* **2007**, *52*, 1171-1178.
3. Chen, Q.; Feng, Y.; Zhang, D.; Zhang, G.; Fan, Q.; Sun, S.; Zhu, D. Light‐triggered self‐assembly of a spiropyran‐functionalized dendron into nano‐/micrometer‐sized particles and photoresponsive organogel with switchable fluorescence. *Advanced Functional Materials* **2010**, *20*, 36-42.
